# A Closer Look at Climate Change Skepticism

**DOI:** 10.1289/ehp.118-a536

**Published:** 2010-12

**Authors:** Charles W. Schmidt

**Affiliations:** **Charles W. Schmidt**, MS, an award-winning science writer from Portland, ME, has written for *Discover Magazine*, *Science*, and *Nature Medicine*

Debate over climate change is nothing new. Scientists have been arguing about whether greenhouse gases released by human activity might change the climate since the late nineteenth century, when Swedish chemist Svante Arrhenius first proposed that industrial emissions might cause global warming.^1^ Fueled by partisan bickering, this dispute now is more bellicose than ever.

Skeptics in the media (typically conservatives) deride global warming as a monumental hoax, while those who believe in the evidence for human-induced climate change (typically liberals) accuse the skeptics of being industry-funded hacks. Meanwhile, efforts to impose cuts on greenhouse gas emissions are failing to get off the ground. Global leaders attending the United Nations Copenhagen Climate Conference in December 2009 were unable to negotiate a binding agreement on how to reduce these emissions.^2^ And despite President Obama’s campaign pledge to make climate change a priority for his administration, bills aimed at transforming U.S. energy policy are stuck in Congress.

But a closer look reveals that what conservative bloggers, pundits, and politicians say about climate change isn’t reflected by what even the more skeptical scientists actually believe. Indeed, scientist-skeptics no longer deny that global warming is actually happening, according to Stefan Rahmstorf, a professor at the Potsdam Institute for Climate Impact Research. Instead, they have shifted their attention to attribution—meaning what’s causing the warming to occur—and whether mandated cuts in greenhouse gases can ever reverse it or should even try.

## Popular Polarity

Claire Parkinson, a climatologist at the National Aeronautics and Space Administration’s Goddard Space Flight Center, claims many scientists who don’t buy into the “mainstream” position on climate change—crystallized by the United Nations Intergovernmental Panel on Climate Change (IPCC) and its view that greenhouse gases are chiefly responsible for what it predicts could be a catastrophic warming of the planet^3^—are reluctant to voice their opinions. “It’s gotten so polarized that scientists who go against the mainstream worry they’ll be treated poorly in the press,” she says. “People will just say, ‘Oh, they’ve been bought off by the oil industry,’ but that’s not always true.”

Ideally, one would like to compare skeptical and mainstream views purely on the basis of science, divorced from political ideology or industry interference. But that’s not always easy, given that scientist-skeptics who do take a public stand often have ties to industry and conservative ideology. For instance, Patrick Michaels, a climatologist who writes skeptical books about global warming, is a visiting scientist at the George C. Marshall Institute, a nonprofit organization sustained in part by oil and gas companies. He also is a fellow at the Cato Institute, a libertarian think tank in Washington, DC. S. Fred Singer, an atmospheric physicist whom many consider to be the godfather of climate change denial, also is linked to numerous conservative and industry organizations.

In 2009 the U.S. Senate Environment & Public Works Committee published a report^4^ listing more than 700^5^ scientist-skeptics expressing a spectrum of dissenting views, many questioning the role of anthropogenic emissions in climate change, although a few are quoted denying climate change altogether. James Inhofe (R–OK), ranking minority member of the committee that produced the report, represents the extreme right wing of his party and has received nearly a million dollars in donations from oil and coal companies since 2000.^6^

The list was compiled by Inhofe’s staff without prior consent by the scientists themselves; Parkinson says some have requested to be taken off the list. Moreover, only 15% of the scientists listed had published in the refereed literature on subjects related to climate science.^7^ Precisely how these individuals line up with respect to their own political views and funding isn’t disclosed in the report and therefore can’t be easily discerned.

In her new book *Coming Climate Crisis?*,^8^ Parkinson argues that industry funding and climate skepticism aren’t necessarily related, however. Those critical of industry funding, she writes, “seem to be claiming that the oil industry wants only one line of results and that the funded scientists are no longer objective.” That’s not always a fair charge, Parkinson points out, especially since some mainstream scientists also take industry funding without comparable criticism. Still, Parkinson warns that all scientists “run a risk when they accept private or corporate funding, especially if the funder is perceived—rightly or wrongly—as seeking results in only one direction.”

## Degrees of Dissent

Parkinson, who says she has never taken money from the fossil fuel industry, says she respects skeptical viewpoints but leans more toward the mainstream view. Given her analysis of the data, she concludes the Earth has, in general, warmed since the dawn of the Industrial Revolution and that greenhouse gas emissions are at least partly to blame. Virtually all scientists agree with at least the first of those conclusions, she says—even the skeptics.

Roger Pielke Sr., a meteorologist and senior research scientist at the University of Colorado, Boulder—who is often associated with the skeptical side of the climate debate but prefers to be called a “climate realist”—agrees. Like Parkinson, Pielke identifies himself as a political independent who doesn’t take funding from the fossil fuel industry. In his view, those who frame the climate change debate as one that pits the IPCC against those who don’t believe global warming is real or that humans have anything to do with it are wrong on both counts. Global warming is happening, he says, and it can’t be explained entirely by natural forces.

Even Michaels concurs. “Of course there’s a warming trend,” he says. “All you have to do is connect the dots. And I can point you to five truly independent papers in world-class journals—not the crackpot stuff you see in unreferenced websites—that must lead you to conclude that slightly less than half of global warming is due to carbon dioxide.”

Mainstream scientists put the blame for climate change almost entirely on greenhouse gases, but scientist-skeptics differ widely in terms of their alternative explanations. Some, such as Tim Patterson, a paleoclimatologist at Carleton University in Ottawa, Canada, emphasize natural “forcings” on the climate, especially solar cycles that affect how much radiation strikes the earth. Others cite man-made influences including industrial emissions of black soot, which warms the air by absorbing sunlight. Still others propose that multiple factors—black soot, land use changes, and more—compound the effects of greenhouse gases on global and regional climate.

Yet acknowledging so many possible causes of climate change leaves policymakers without any obvious solutions. And whereas mainstream scientists believe reducing greenhouse emissions is the key, skeptics aren’t unified around any alternate strategy. However, at least one—Pielke—supports a modest, politically acceptable carbon tax to fund alternative energy research.^9^

## Economic Implications

The scientific debates on climate change have massive economic implications, which explains why a disagreement that would ordinarily be worked out in the peer-reviewed literature has created such a polarized social divide. Attempts to reverse climate change could inflict enormous costs on industries that will fight to the death for their survival.^10^

Bjørn Lomborg, a Danish writer and author of *The Skeptical Environmentalist*,^11^ argues that if imposed today, efforts to reduce greenhouse gas emissions by 80% by 2050 (one goal of the Obama administration) would cost trillions of dollars and inflict more pain than climate change itself. Not surprisingly, governments won’t agree to those costs, he says, which is why international meetings like the Copenhagen conference fail every time they’re held.

“One of the definitions of insanity is doing the same thing again and again and expecting a different outcome,” Lomborg says. “At some point, you have to ask yourself if you need a different approach.”

Lomborg agrees with the IPCC’s view that greenhouse gases account for most of the temperature increases of the last 100 years,^3^ but he rejects mandating emissions cuts now. Those cuts, he says, would have to be made using green technologies that aren’t yet cost-effective. Moreover, $1 invested now would save only 2¢ in future climate change damage.^12^ Instead, Lomborg advocates for massive investments in green technology—citing research by McGill University’s Chris Green,^13^ he says this could produce more cost-effective emission reductions later, with $1 invested saving around $11 in future climate change damage.

## Snowball Effect

Unfortunately, these nuances get lost in the extreme rhetoric on climate change playing out in the mass media. Instead of engaging in cool-headed discussions, some mainstream sympathizers attack anyone who disagrees with the IPCC, while conservatives cherry-pick isolated events and statements to undermine the evidence for global warming.

That was plainly evident late last year, when scientists discovered the IPCC had erroneously stated that all 15,000 Himalayan glaciers could melt by 2035, which is implausible under even the worst climate scenarios, according to Jeffrey Kargel, the glaciologist at the University of Arizona who first noticed the error. That error was ultimately found to be a clerical mistake (the correct year was supposed to be 2350, Kargel says) appearing first in *New Scientist* magazine,^14^ then in a World Wildlife Fund brochure,^15^ and finally in the IPCC’s Fourth Assessment Report,^3^ released in 2007.

The media response was nothing short of extraordinary. Every major newspaper in the world reported the error, which dealt a blow to the IPCC’s reputation. Kargel says that’s unfortunate. “This was a very embarrassing, grossly erroneous paragraph in an encyclopedic document,” he wrote in a 22 January 2010 response to an article in *The Economist*.^16^ “The IPCC Fourth Assessment is 99.9% correct, as far as science knows . . . and almost all media reporting in the last week is not about that 99.9%; it’s about the 0.1%.” Yet the incident also highlighted the fact that the Fourth Assessment had based this particular conclusion at least in part on “gray literature”—non-peer-reviewed or nonpublished sources.

The IPCC suffered yet another embarrassment when nearly 1,000 hacked e-mails originating from the University of East Anglia’s Climatic Research Unit (CRU) in Norwich, UK, went viral on the Internet. The CRU is one of the IPCC’s main suppliers of trend data on global temperatures. Several exchanges between CRU director Phil Jones and other scientists suggested they were trying to block dissenting evidence from the peer-reviewed literature. During the resulting furor, dubbed “Climategate,” Jones stepped down temporarily from his position as CRU director but was reinstated in July 2010. Skeptical media have since voiced the view that just a few influential scientists control the IPCC and squelch dissenting views.

## The Viewing Public: Caught in the Crossfire

These events may be exacerbating declines in public confidence about the evidence for climate change. A 2010 poll by the Yale School of Forestry & Environmental Studies found that 57% of respondents answered yes to “Do you think that global warming is happening?” compared with 71% in 2008.^17^ Similar results were obtained in Great Britain by Nicholas Pidgeon, a professor at Cardiff University. Pidgeon found the number of British respondents who answered yes to the question “As far as you know, do you personally think the world’s climate is changing, or not?” dropped from 91% in 2005 to 78% in 2010.^18^

Pidgeon blames the decline on a number of factors, including “issue fatigue” and a financial crisis that for many has become a bigger worry. “But climate-skeptic agendas [within politics] are also becoming more prominent,” he says. “You have a lot of groups and individuals engaging in long-standing attempts to highlight uncertainties in the science.”

Because scientists are faced with the unenviable task of informing policy decisions on climate change, Parkinson advises caution in how they communicate with the media. “Scientists might get flustered and say things they could have said better with a little more forethought,” she says. “You hear exaggerations like ‘once sea ice retreats, it can’t come back,’ which is absurd. Of course ice can come back. Or you might hear a scientist say ‘all glaciers are retreating,’ which is, of course, false—many are retreating, but some aren’t. As soon as you make an ‘all-or-none’ statement like that, you open yourself up to an attack from someone on the other side, and then you’re trying to defend your credibility.”

## Figures and Tables

**Figure:**
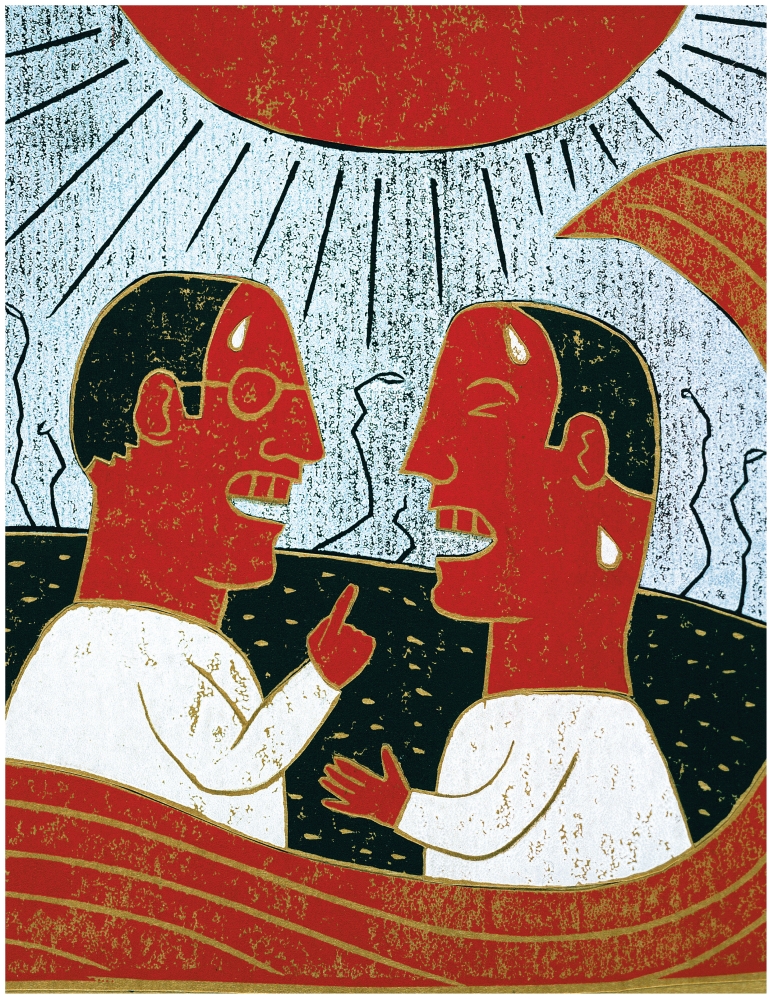


**Figure:**
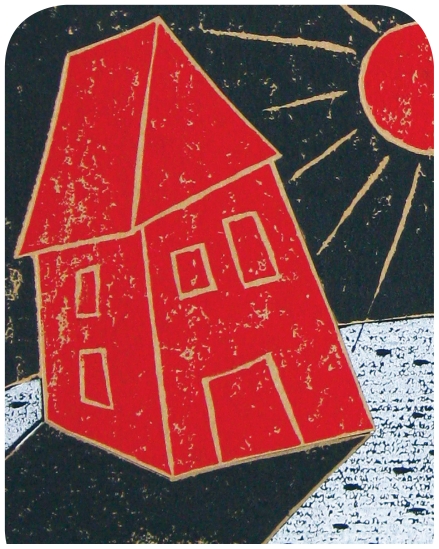
Those who frame the climate change debate as one that pits the IPCC against those who don’t believe global warming is real or that humans have anything to do with it are wrong on both counts, according to Roger Pielke Sr. Global warming is happening, he says, and it can’t be explained entirely by natural forces.

**Figure:**
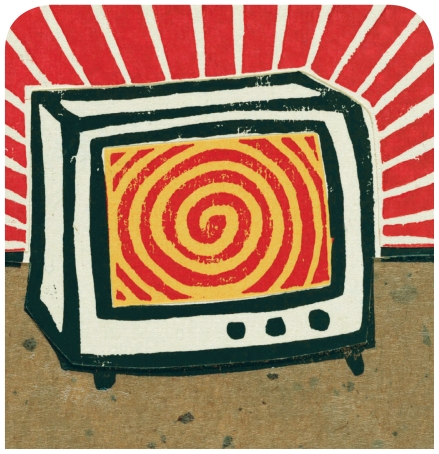
The nuances of the climate change discussion get lost in the extreme rhetoric playing out in the mass media. Instead of engaging in cool-headed discussions, some mainstream sympathizers attack anyone who disagrees with the IPCC, while conservatives cherry-pick isolated events and statements to undermine the evidence for global warming.

**Figure:**
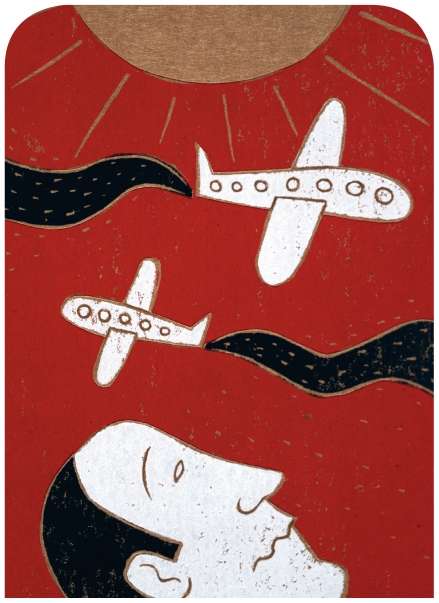
The political debate over climate change may be exacerbating declines in public confidence in the scientific evidence. One U.S. poll found that 57% of respondents answered yes to “Do you think that global warming is happening?” in 2010 compared with 71% in 2008. A British poll found that only 78% of respondents answered yes to the question “As far as you know, do you personally think the world’s climate is changing, or not?” compared with 91% in 2005.
